# Common in Adults and Often Overlooked in Pediatrics: A Case Report of Primary Hyperparathyroidism in an Adolescent Patient

**DOI:** 10.7759/cureus.38112

**Published:** 2023-04-25

**Authors:** Elizabeth Boggs, John Szymusiak

**Affiliations:** 1 Internal Medicine, University of Colorado School of Medicine, Aurora, USA; 2 Internal Medicine-Pediatrics, University of Pittsburgh Medical Center, Pittsburgh, USA

**Keywords:** multiple endocrine neoplasias, hypercalcemia, primary hyperparathyroidism, adolescent, pediatric

## Abstract

Primary hyperparathyroidism (pHPT) is a rare clinical entity in pediatric patients relative to adults. Consequently, the diagnosis is often delayed in pediatric patients, and children and adolescents are more likely to present with symptoms of hypercalcemia and end-organ damage. Here, we present the case of an adolescent patient with chest pain who was found to have a lytic bone lesion secondary to pHPT.

## Introduction

Primary hyperparathyroidism (pHPT) is a broad term that encompasses sporadic isolated parathyroid adenoma, parathyroid hyperplasia related to multiple endocrine neoplasias (MEN), and familial non-MEN pHPT. In both pediatric and adult patients, parathyroid adenoma is the most common cause of pHPT, although the incidence of hereditary pHPT is higher among pediatric patients than adults [1,2].

pHPT is rare in the pediatric population, affecting only about two to five out of every 100,000 children [1]. In contrast, roughly one in every 1,000 adults is affected by pHPT. As a result, pHPT is often neglected in the differential diagnosis of pediatric patients presenting with nonspecific symptoms such as fatigue, depression, nausea, musculoskeletal pain, and headache, leading to delayed diagnosis and increased risk of end-organ damage [1-4]. Interestingly, the incidence of pHPT in pediatric patients has been increasing over the past few decades, likely due to the more widespread use of electrolyte testing and the wider availability of parathyroid hormone assays [5].

A version of this article was presented as a poster at the 2016 American Academy of Pediatrics National Conference and Exhibition in October 2016.

## Case presentation

**Figure 1:**
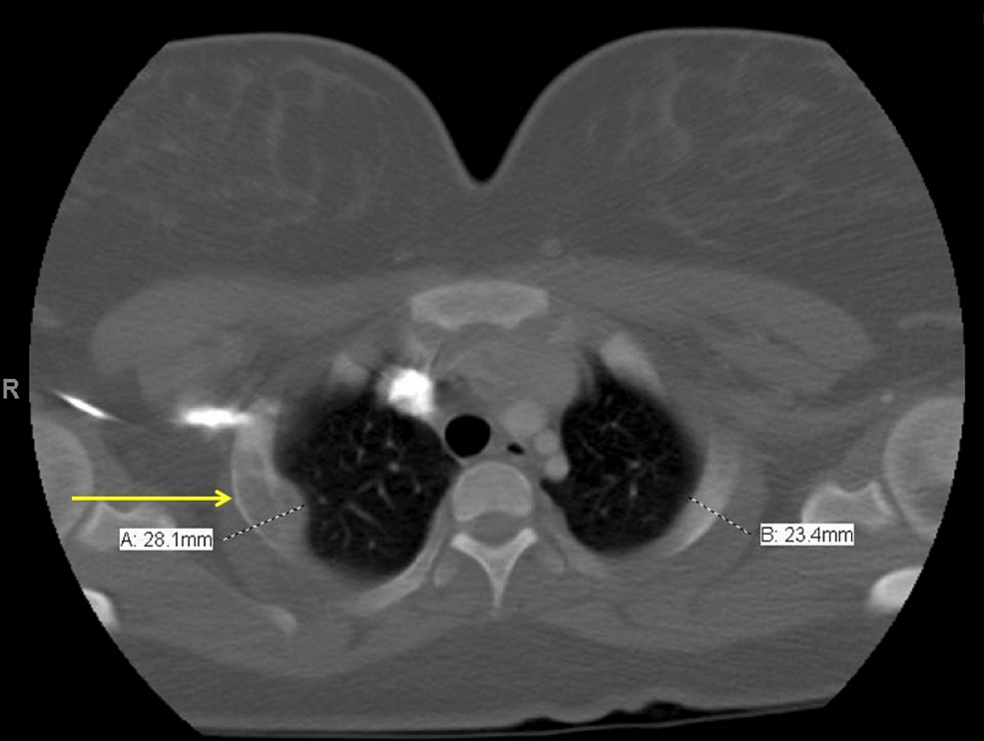
Chest CT with a focal lytic lesion of the posterolateral right second rib (arrow) with small extra-pleural-based soft tissue density. CT, computed tomography

An 18-year-old female with a past medical history of mild intermittent asthma and a family history of unspecified thyroid carcinoma presented to the emergency department with a two-day history of pleuritic chest pain and shortness of breath. Vital signs were within normal limits, and cardiovascular and respiratory exams were unremarkable. The musculoskeletal exam was notable for mild tenderness to palpation of the right parasternal region. The initial workup for chest pain and shortness of breath in this patient included normal ECG and a chest X-ray with a focal lytic lesion of the posterior lateral right second rib with small extra-pleural-based soft tissue density. Follow-up chest CT with contrast showed a lytic lesion involving the posterior part of the right second rib with focal cortical disruption and small extra-pleural soft tissue overall concerning Ewing's sarcoma (Figure 1). A heterogeneous nodule with a fatty component in the left thyroid lobe was also noted.

Given the concern for malignancy, lab work was obtained for further diagnostic workup. CBC was within normal limits. The comprehensive metabolic panel was significant for mildly elevated total calcium (11.4 mg/dL; range 8.5-10.2), a normal serum albumin and protein, low phosphorus (1.9 mg/dL; range 4.5-6.5), and elevated alkaline phosphatase (247 International Unit (IU)/L; range 30-180). The patient was admitted to the Hematology/Oncology service for further workup of possible malignancy.

Subsequent testing revealed supraphysiological elevated parathyroid hormone (PTH; 953 pg/mL; range 10-65). Thyroid function tests, thyroglobulin level, and prolactin level were all normal. Both the rib lesion and thyroid nodule were biopsied by interventional radiology to rule out sarcoma. The rib biopsy was nondiagnostic, and the thyroid nodule pathology showed parathyroid tissue consistent with the parathyroid cyst.

**Figure 2:**
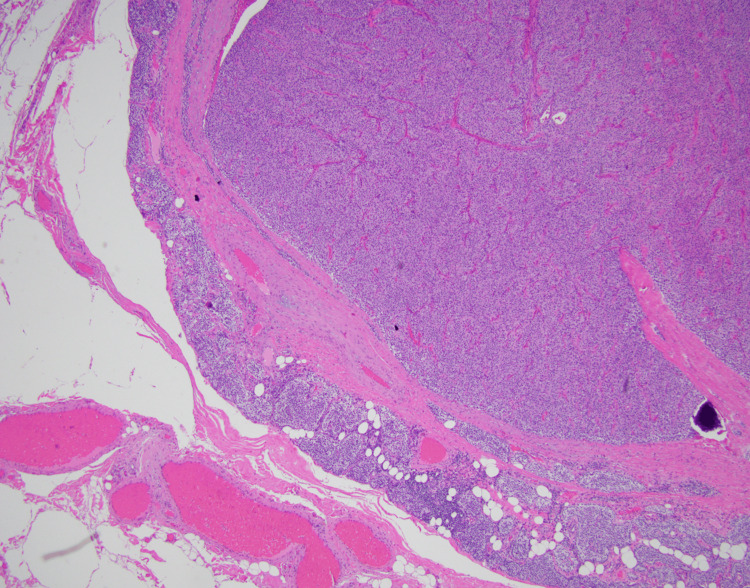
Pathology from L interior parathyroidectomy showing the hypercellular L inferior parathyroid gland with cystic degeneration consistent with hyperfunctioning tissue.

The patient ultimately underwent left inferior parathyroidectomy. Pathology showed an enlarged, hypercellular left inferior parathyroid gland with cystic degeneration, consistent with probable hyperfunctioning tissue (Figure 2). Postoperatively, the PTH level decreased from 573 to 31 pg/mL. The patient developed profound hypocalcemia and hypophosphatemia consistent with the hungry bone syndrome, a common complication of parathyroidectomy in patients with preexisting osteopenia due to the sequestration of calcium and phosphate in bone in the immediate postoperative period. This was resolved with aggressive calcium and phosphorus supplementation. Genetic workup for multiple endocrine neoplasia syndromes was negative. Given these findings, ultimately, the patient was diagnosed with pHPT complicated by a lytic bone lesion of the right rib.

## Discussion

pHPT, a disease that typically affects adults over the age of 50 to 65 years, was diagnosed in this female adolescent who presented with chest pain and shortness of breath. Given the comparatively low incidence of parathyroid disorders in pediatric patients, physicians frequently fail to check serum calcium levels when evaluating children with nonspecific complaints. This leads to a delay in the recognition and diagnosis of pediatric parathyroid disorders and can increase the risk for end-organ pathology. As a result, pediatric patients are much more likely than adults to present with symptomatic pHPT. Studies indicate that roughly 80% to 90% of pediatric patients were symptomatic at the time of diagnosis, in comparison to 20% to 50% of adult patients, who most often present with asymptomatic hypercalcemia [1]. The most common symptoms of pHPT among pediatric patients at the time of diagnosis include fatigue, headache, nephrolithiasis, gastrointestinal symptoms, bone disease, or pancreatitis [1,3]. Nephrolithiasis, bone involvement, and nephrocalcinosis are among the most common end-organ pathologies in pediatric patients. Bone disease associated with pHPT includes bone pain, osteopenia, fractures, or lytic bone lesions [3].

Diagnosis of pHPT in both adults and children begins with assessing serum calcium and parathyroid hormone levels. In adult patients, alkaline phosphatase is a useful prognostic indicator of bone involvement and requirement for postoperative calcium supplementation due to hungry-bone syndrome. However, the relationship between alkaline phosphatase and bone involvement in pediatric patients is less clear given the wider range of normal alkaline phosphatase levels in children and physiologic variation based on age [1].

Prior studies suggest that 80% of pediatric patients presenting with a single parathyroid adenoma have pHPT. Patients with MEN and familial non-MEN HPT are more likely to present with multiple adenomas; only about 10% of these patients present with a single adenoma. This suggests that diagnostic workup to rule out inherited endocrinopathies may not be necessary for older adolescents with no family history of inherited pHPT who present with single parathyroid adenoma.

As in adults, surgical therapy is highly effective in lowering serum calcium but may not reverse end-organ damage in pediatric patients. Single gland parathyroidectomy, as in this case, is effective for isolated adenoma and should be the standard approach, given the low overall prevalence of multiple gland disease in pediatric patients without a family history of the parathyroid disease [6,7]. Subtotal parathyroidectomy or total parathyroidectomy with autotransplantation is used to treat MEN and familial non-MEN pHPT hyperplasia [1].

It should be noted that pediatric patients may experience higher rates of treatment failure after the primary operation (20%) in comparison to adults (1%) [1]. The limited literature on the topic argues for the routine use of ultrasonography with or without sestamibi scanning before surgical exploration in children [3]. The increased rate of postoperative treatment failure in children is thought to be due to a higher incidence of ectopic parathyroid glands in the pediatric population [3].

## Conclusions

pHPT is a rare disease entity in the pediatric population, although it is more common in adolescents than in younger children. Pediatric patients are more likely than adults to have symptoms at the time of diagnosis and are more likely than adults to experience end-organ damage due to delayed diagnosis. Pediatricians should keep pHPT as part of their differential in pediatric patients presenting with nonspecific complaints and consider checking a calcium level as part of the workup for these patients. pHPT should also be on the differential diagnosis in pediatric patients who present with lytic bone lesions. When HPT is diagnosed in a pediatric patient, a genetic workup for multiple endocrine neoplasia syndromes is lower yield in adolescents with no family history of endocrinopathies. As in adults, solitary adenomas are the most common underlying etiology of pHPT and are effectively treated with surgical intervention, although providers should consider evaluation for ectopic parathyroid tissue in pediatric patients before surgery.
